# Deformation and rupture of microcapsules flowing through constricted capillary

**DOI:** 10.1038/s41598-021-86833-8

**Published:** 2021-04-08

**Authors:** Bruna C. Leopércio, Mariano Michelon, Marcio S. Carvalho

**Affiliations:** 1 Laboratory of Microhydrodynamics and Flow in Porous Media, Department of Mechanical Engineering, Pontifical Catholic University of Rio de Janeiro, Rio de Janeiro, RJ 22451900 Brazil; 2 School of Chemistry and Food Engineering, Federal University of Rio Grande, Rio Grande, RS 96203900 Brazil

**Keywords:** Engineering, Materials science, Physics

## Abstract

The dynamics of deformable microcapsules flowing through constricted channels is relevant in target delivery of chemicals in physiological systems, porous media, microfluidic medical diagnostic devices and many other applications. In some situations, the microcapsules need to sustain the stress they are subjected to as they flow through constricted channels and in others, the stress may be the rupture trigger used to release the internal content. We experimentally investigate the flow of monodispersed gellan gum microcapsules through a constricted capillary tube by measuring the evolution of the pressure difference and flow visualization. The maximum pressure difference and capsule deformation is obtained for capsules with different diameter and shell thickness. We map the conditions, e.g. diameter and shell thickness, at which the capsule membrane ruptures during the flow, releasing its internal phase.

## Introduction

Microcapsules are formed by a fluid core enclosed by a solid flexible shell. The shell can be made of different materials and its main function is to protect the inner content and, in most applications, control its release^1–3^. Microcapsules have a broad range of applications, including in food^4–6^, cosmetic^7,8^, pharmaceutical industries^9,10^ and reservoir engineering^11–13^. In most of these applications, microcapsules are used to transport a desired chemical component, avoiding interaction with the environment, to a specific region where it should be released at a specific time. Microcapsules can also be used as a means to control the mobility of a water phase injected in a reservoir, partially blocking the pore throats and diverting water to oil-filled.

Controlling the encapsulation process and release characteristics of the microcapsules is essential for an adequate application. This ultimately depends on the technique and material chosen to produce the microcapsules. Emulsification technology is a key step in microencapsulation once monodispersed microcapsules are often made from single or double emulsion drops. Thus, microfluidics has appeared as an effective technique for the fabrication of monodisperse double emulsion templates that are subsequently converted into microcapsules with well-controlled release properties. It enables tuning the template dimensions and using a broad range of materials to form the microcapsule shell^14,15^. The controlled content delivery can happen as a consequence of mechanical rupture, biodegradation, dissolution or melting of the shell or diffusion through it^16^. Several triggers can be used to start the release process, depending on the shell material. The most common are temperature^17,18^, pH^19^, osmotic pressure^20,21^, chemical reaction^22,23^ and external stress^22^.

In many applications, microcapsules flow through confined geometries, which are present in physiological systems and porous media, making the dynamics of deformable microcapsules in confined flows relevant to the success of target chemical delivery by a microcapsule suspension. In some cases, it is important to design microcapsules that can sustain the stress and deformation that they are subjected to as they flow through constricted channels, such that the encapsulated chemical can reach the target region. On the other hand, there are situations in which the content release is triggered by an external stress, which can be achieved during the flow through a constriction^22^.

Microcapsule is widely considered a good model to describe the hydrodynamic behavior of cells^24^, and the flow of suspended microcapsules through confined channels has attracted much attention also because of the development of microfluidic medical diagnostic devices based on cell deformation as they flow through constricted capillaries^25,26^. Healthy and diseased cells have different stiffness and the dynamics of deformation can be used to separate normal and cancer cells, for example^27^.

Flow of suspended microcapsules through confined channels poses a challenging problem due to its complex fluid–solid interaction. A myriad of experimental and numerical studies has been developed to understand the underlying physical phenomena and were discussed in a recent review^28^.

The flow of suspended capsules through confined geometry has been extensively studied by numerical simulation. Leyrat-Maurin and Barthès-Biesel^29^ presented a model for the flow of a capsule through an axisymmetric hyperbolic constriction, considering both a constant flow rate and constant pressure-drop condition to infer properties of the capsules from filtration experiments. Details of the capsule deformation and extra pressure-drop as the capsule flows through the constriction were determined as a function of capsule properties and flow conditions. The three-dimensional flow of an elastic capsule through constricted channels has been studied to relate the capsule deformation and extra pressure with the membrane elastic properties and flow conditions^30,31^. From their computational predictions, Dimitrakopoulos and Kuriakose^31^ proposed a method based on the flow in a converging micro capillary to evaluate the membrane shear modulus independently of its dilation modulus.

Experimental analysis of microscale flow of suspended microcapsules is challenging. Risso et al.^32^ experimentally studied motion and deformation of bioartificial capsules in a 4 mm diameter capillary. They showed that the steady-state capsule configuration was a function of the capillary number, defined as the ratio of viscous to elastic forces, and the capsule diameter to tube radius ratio. The capsule deformation in the flow through microchannels of comparable dimensions was also used by Lefebvre et al.^33^ to characterize the membrane mechanical properties of microcapsules. The transient flow of ovalbumin microcapsules through convergent-divergent square microchannels, with cross section area varying from 30 × 30 to 70 × 70 μm^2^, was studied by Leclerc et al.^34^. The relaxation process of the capsule back to its nearly undeformed configuration as it flows out of the constricted channel was used to extract the shear modulus of the membrane. Flow through a convergent channel was also used to evaluate elastic properties of cells enabling cell sorting and cancer diagnostics^35^. Most of the experimental analyses are focused on the determination of the capsule membrane properties and do not report the extra pressure associated with the flow of the capsule through a constriction, which would be an important parameter to validate available numerical predictions. The effect of shell stiffness and thickness of PDMS capsules on the pressure difference of the flow through constricted capillary was studied by do Nascimento et al.^36^. PDMS capsules are strong enough that capsule burst was not observed in any condition. As discussed in the review by Barthès-Biesel^28^, there are very few observations of flow induced capsule burst; the capsules used in the experiments have been too resistant or the flow strength too weak to rupture the capsule membrane.

This work reports the evolution of the deformation and pressure difference in the flow of a suspended soft microcapsule through a constricted microcapillary in a constant flow rate condition. We discuss the effect of capsule size and shell thickness on the flow mobility and shell rupture.

## Results and discussion

The dimensionless parameters that govern the flow of a suspended capsule through a constricted capillary are^29^:

1. Dimensionless capsule diameter: $\overline{a} = \frac{D}{{D_{c} }}$,
2. Capillary constriction ratio: $\beta = \frac{{D_{c} }}{{D_{0} }}$,
3. Surface capillary number: $Ca_{s} = \frac{{\mu_{o} \overline{V}}}{Gh}$,
4. Reynolds number: $$Re = \frac{{\rho \overline{V}D_{o} }}{{\mu_{o} }}$$,
5. Viscosity ratio: $$\lambda = \frac{{\mu_{i} }}{{\mu_{o} }}$$,

where $D$ is the capsule diameter, $h$ is the shell thickness, $D_{0}$ and $D_{c}$ are the capillary and constriction diameter; $\overline{V}$ is the average flow velocity; $G$ is the shear modulus of the shell material; and $\mu_{i}$ and $\mu_{o}$ are the viscosity of the inner and outer phases.

The surface capillary number represents the ratio of viscous to elastic forces. High surface capillary number indicates that viscous force, which deforms the capsule, is larger than the resisting membrane elastic force and leads to highly deformed capsules.

In the present analysis, the capillary geometry, Reynolds number and viscosity ratio were fixed at $\beta = 0.33, Re = 0.021$ and $\lambda = 4.81$, respectively. The dimensionless capsule diameter $\overline{a}$ and the surface capillary number $Ca_{s}$ were varied by changing the capsule diameter and shell thickness. Batches of gellan microcapsules with different diameter $D$ and shell thickness $h$ were produced by microfluidics and used in the experiments (see Table 1). The diameters and shell thicknesses presented are the mean value of 10 measurements made for each batch with their standard deviation. All capsules were smaller than the capillary diameter and larger than the constriction diameter through which they flowed.

**Table 1:** Main properties of the microcapsules used in the experiments.

System ID	$D$ (μm)	$h$ (μm)
#1	174.6 ± 4.6	15.0 ± 2.1
#2	143.8 ± 4.2	6.3 ± 1.5
#3	144.4 ± 6.7	15.2 ± 3.7
#4	124.4 ± 1.7	5.6 ± 1.0
#5	109.1 ± 2.9	5.3 ± 1.1

**Figure 1:**
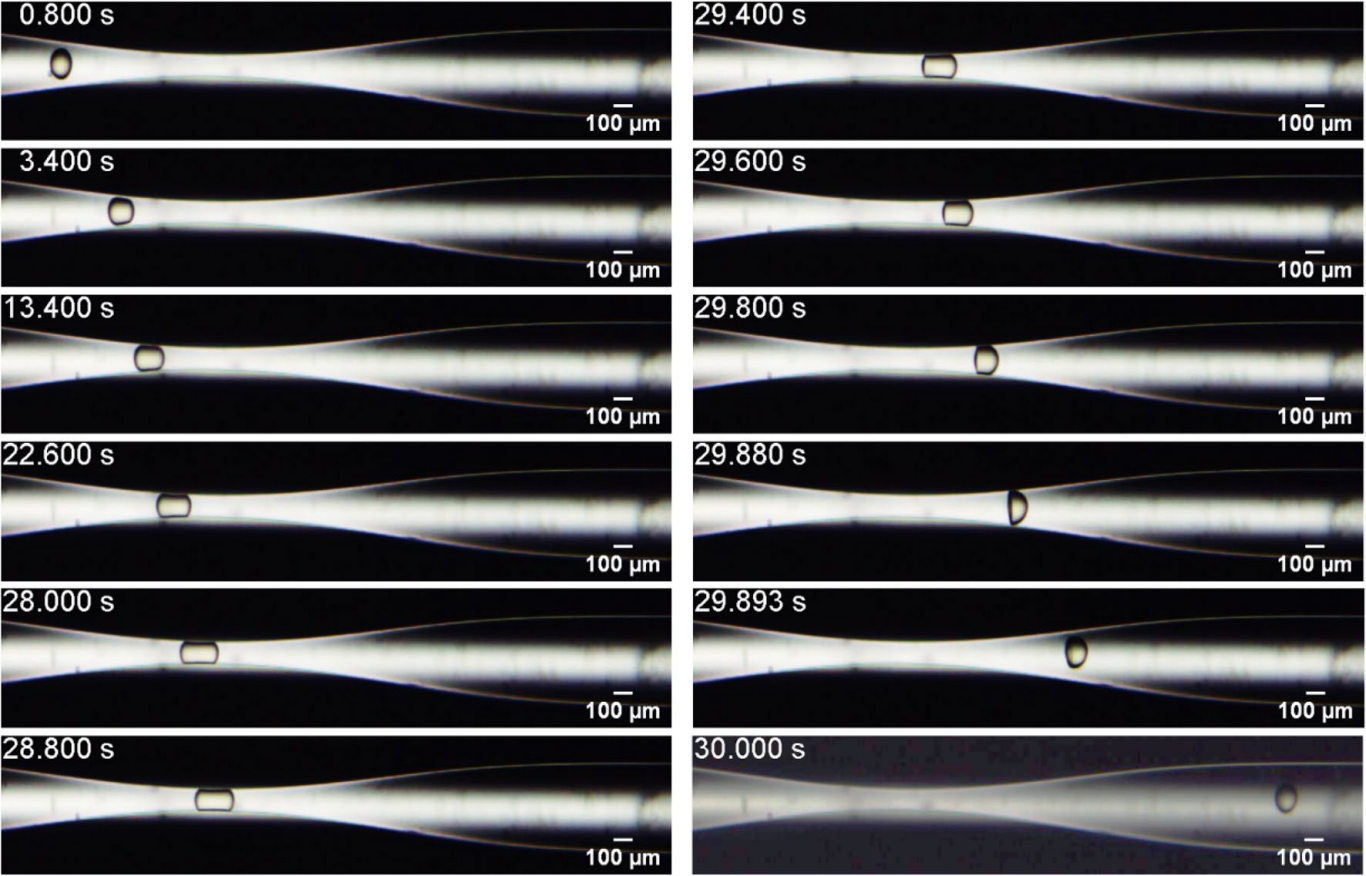
Evolution of the microcapsule position and configuration as it flows through the constriction. System #3, $D = 144.4{ }$ μm and $h = 15.2$ μm $(\overline{a} = 1.44$and $Ca_{s} = 5.9 \times 10^{ - 4}$).

**Figure 2:**
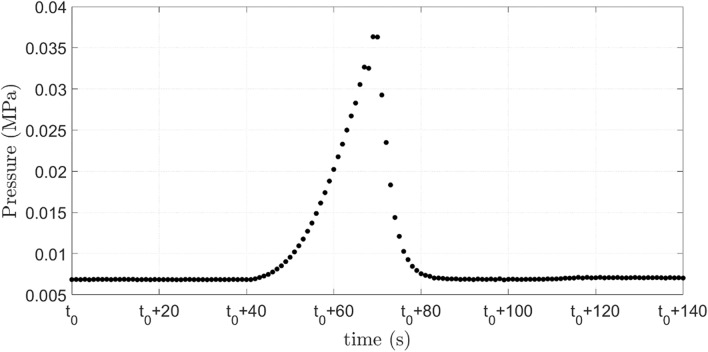
Evolution of the inlet pressure as the capsule flows through the constriction. System #3, $D = 144.4{ }$ μm and $h = 15.2$ μm $(\overline{a} = 1.44$ and $Ca_{s} = 5.9 \times 10^{ - 4}$).

Figure 1 presents snapshots of the flow as a capsule pass through the constriction for System #3, with $D = 144.4$ μm and $h = 15.2{ }$ μm (supplementary video S1), which corresponds to $\overline{a} = 1.44$ and $Ca_{s} = 5.9 \times 10^{ - 4}$. The capsule does not burst under these conditions. The capsule partially blocks the flow, causing an increase in the inlet pressure, as shown in Fig. 2. When the capsule is far from the capillary throat, the inlet pressure is close to the one necessary to drive the continuous phase alone, e.g. approximately $5.4 \times 10^{ - 3} {\text{MPa}}$. The inlet pressure grows in order to force the microcapsule deformation, reaches a maximum value of approximately $35.8 \times 10^{ - 3} {\text{MPa}}$ and then abruptly falls as the microcapsule flows away from the constriction. When the capsule is far downstream from the throat, the inlet pressure reaches the steady state associated with the flow of the continuous phase alone. Experiments were repeated for at least 5 different capsules of each system. The behavior for each capsule of a same system was the same. For capsules of System #3, the standard deviation of the pressure peak as the capsule flows through the constriction was close to 11%.

**Figure 3:**
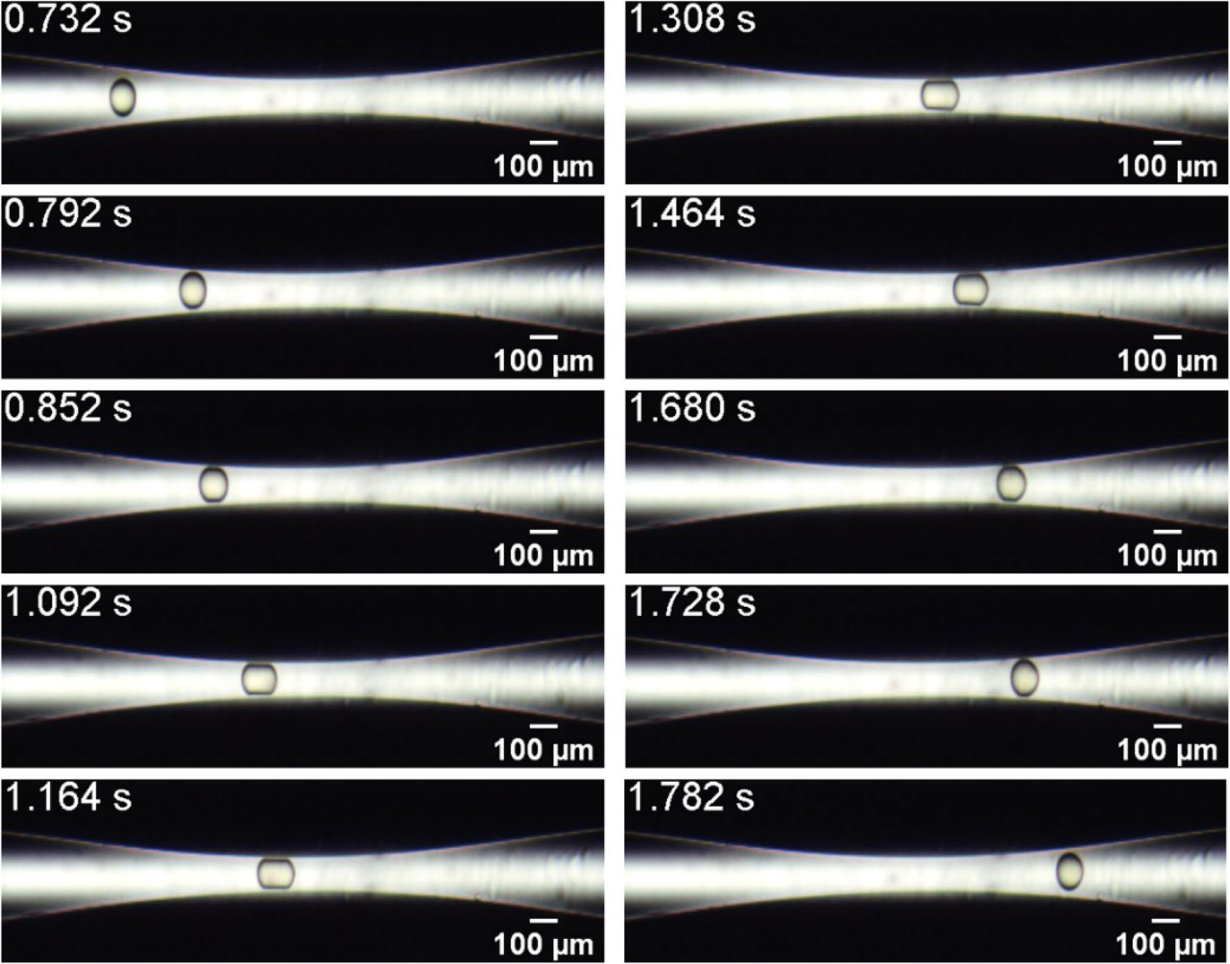
Evolution of the microcapsule position and configuration as it flows through the constriction. System #5, $D = 109.1{ }$ μm and $h = 5.3$ μm ($\overline{a} = 1.09$ and $Ca_{s} = 1.7 \times 10^{ - 3} )$.

Figure 3 presents snapshots of the flow of a capsule from System #5, $D = 109.1{ }$ μm and $h = 5.3{ }$ μm, which corresponds to $\overline{a} = 1.09$ and $Ca_{s} = 1.7 \times 10^{ - 3}$. At these conditions, the capsule also does not burst as it passes through the constriction (supplementary video S2). Microcapsules from this system are smaller than the ones from System #3 (example shown in Fig. 1) and thereby pass more easily and faster through the capillary throat. The extra pressure associated with the flow of the capsule through the constriction could not be measured because it was below the transducer resolution ($2.07{ } \times 10^{ - 3} {\text{ MPa}}$).

**Figure 4:**
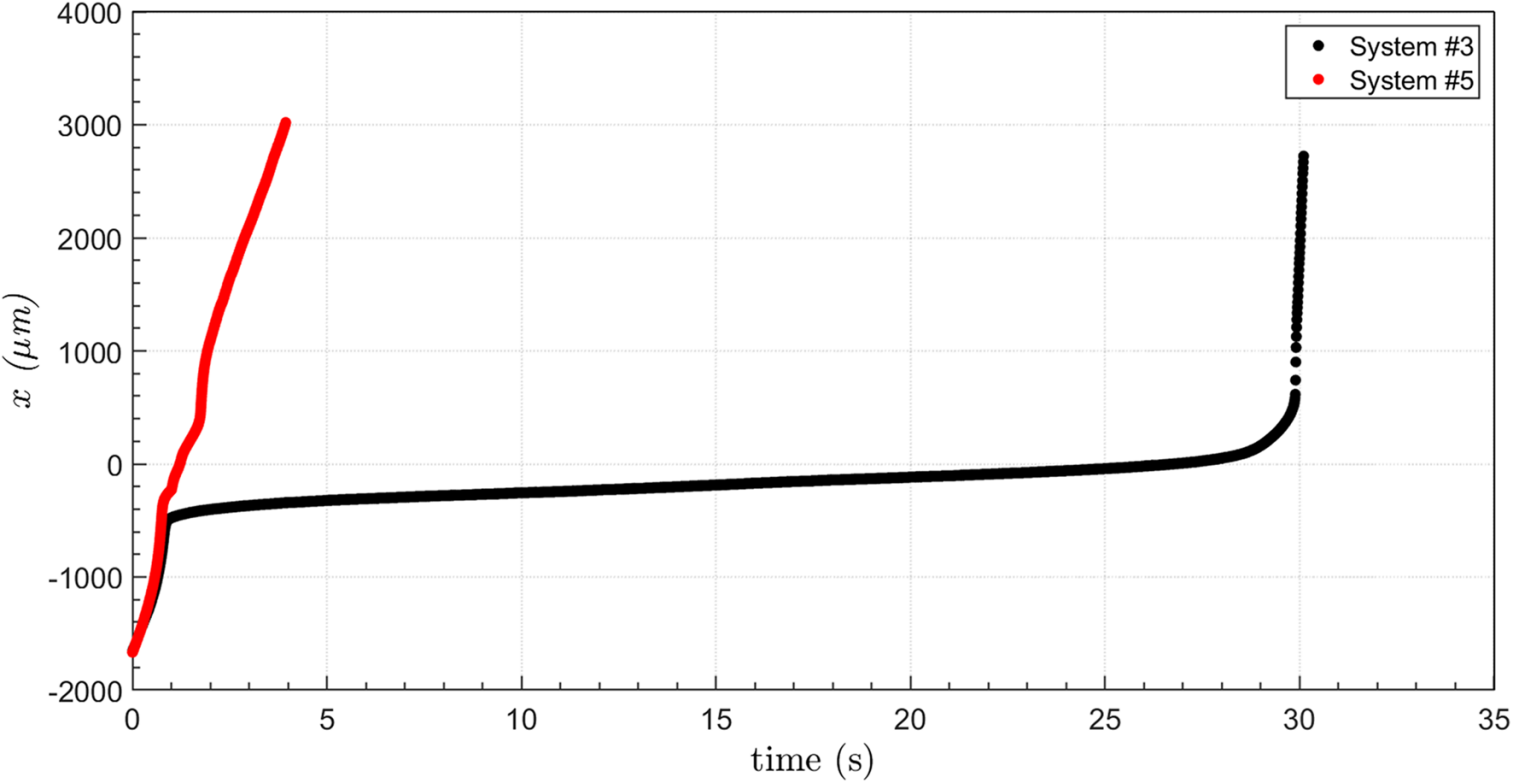
Microcapsule position in the flow direction as it flows through the constriction for capsules from System #3 $(\overline{a} = 1.44$ and $Ca_{s} = 5.9 \times 10^{ - 4}$) and System #5 $(\overline{a} = 1.09$ and $Ca_{s} = 1.7 \times 10^{ - 3}$).

The evolution of the capsule tip position in the flow direction $$x$$ for capsules from System #3 and #5 is shown in Fig. 4. Far upstream of the minimum diameter position ($$x = 0)$$, capsules from both systems present the same position evolution, since the imposed flow rate is fixed for all experiments. For capsules from System #3, which have larger diameter, the velocity is drastically reduced as it deforms to flow through the constriction. The capsule slows down approximately 500 μm upstream of the minimum diameter plane ($$x = 0$$). It stays near the constriction during approximately 26 s, with an average speed close to $$v = 14$$ μm/s. During this period, the inlet pressure grows, as shown in Fig. 2. As it deforms and flows through the constriction, its velocity is reduced to approximately $$v = 825{ }$$ μm/s (from $$t \approx 0.8{\text{ s to t}} \approx 1.7{\text{ s}})$$. Then, the microcapsule is quickly expelled from the constriction and flows away from it.

**Figure 5:**
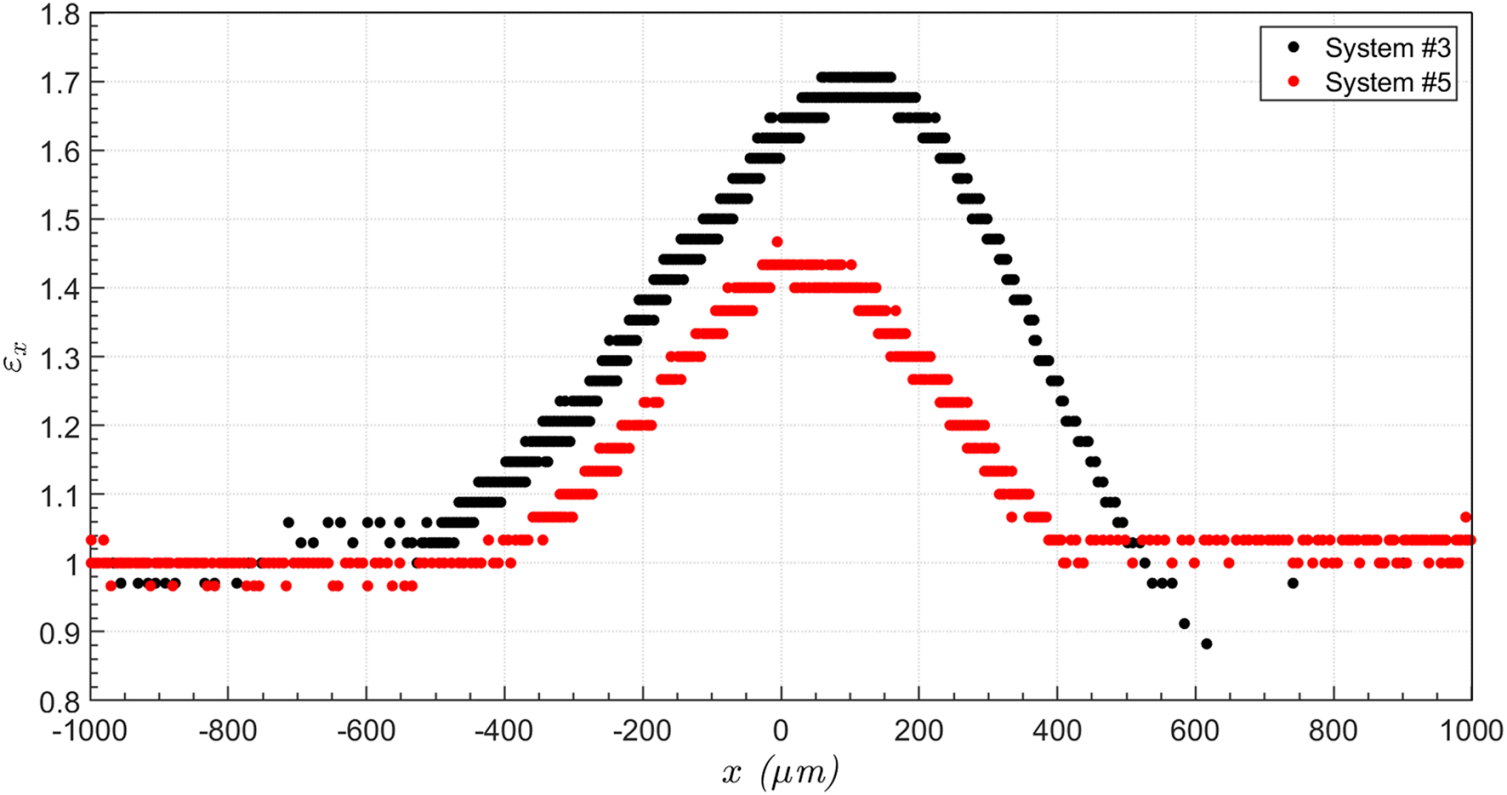
Microcapsule deformation $\varepsilon_{x} = L_{x} /L_{x,0}$ as it flows through the constriction for capsules from System #3 $(\overline{a} = 1.44$ and $Ca_{s} = 5.9 \times 10^{ - 4}$) and System #5 $(\overline{a} = 1.09$ and $Ca_{s} = 1.7 \times 10^{ - 3}$).

As the capsules’ diameters are larger than the throat diameter, the capsules deform as they flow through the constriction. The deformation may be quantified by the ratio of the length of the capsule at a given instant of time to the length of the undeformed capsule, away from the constriction, e.g. $\varepsilon_{x} = L_{x} /L_{x,0}$. Figure 5 presents the evolution of the deformation $\varepsilon_{x}$ for capsules from System #3 and #5. Away from the constricted region of the capillary, $\varepsilon_{x} \approx 1$, as expected. As the capsule approaches the throat, $\varepsilon_{x}$ rises to a maximum value of approximately $\varepsilon_{x} \approx 1.71$ for System #3 and $\varepsilon_{x} \approx 1.42$ for System #5. Downstream of the throat, the larger capsules (from System #3) presented a backend with a concave configuration, leading to values of $\varepsilon_{x} < 1$, which has been observed in some numerical analyses^29,30^, before relaxing back to its nearly undeformed state.

**Figure 6:**
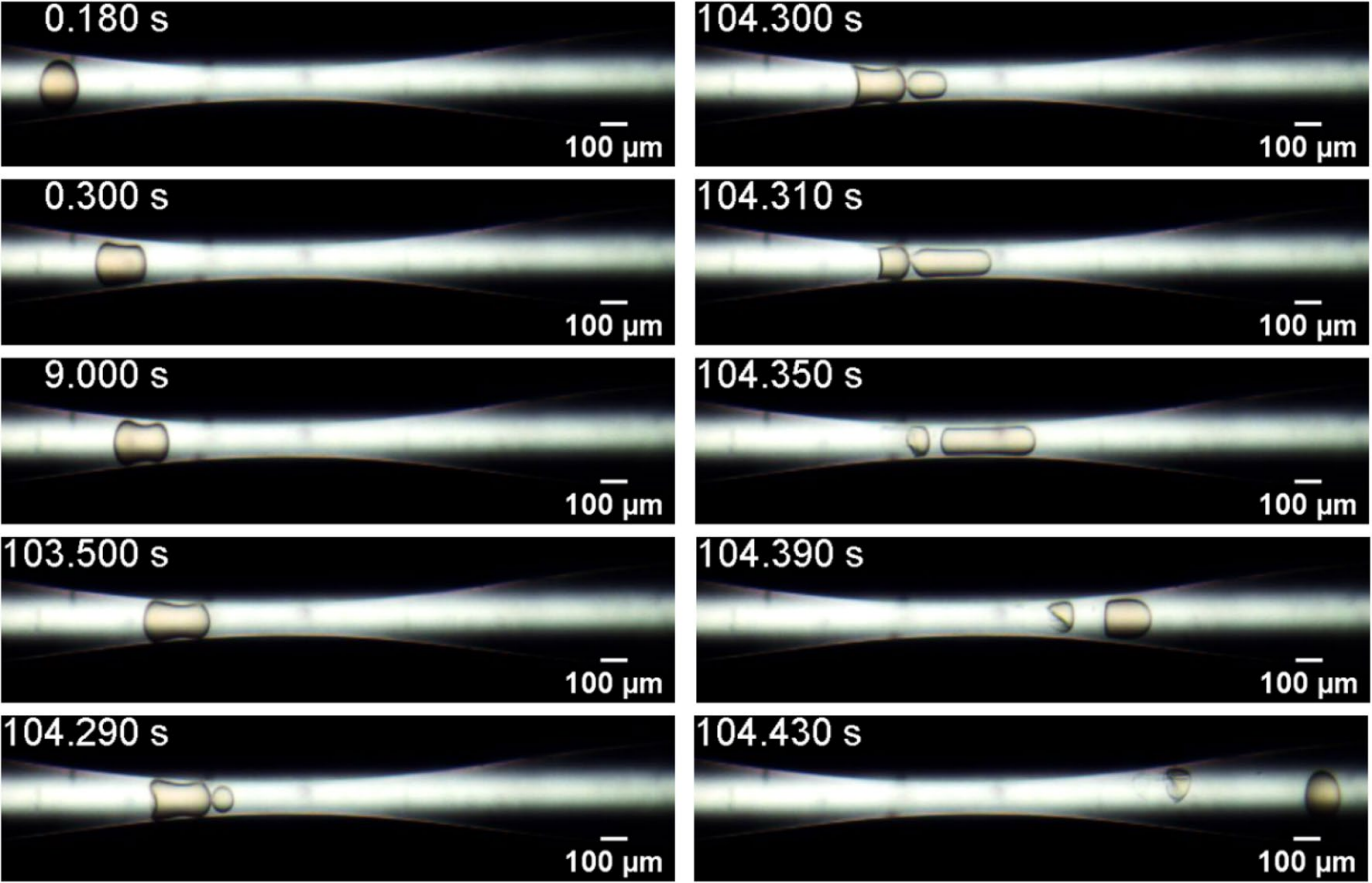
Evolution of the microcapsule position and configuration as it flows through the constriction. System #1, $D = 174.6{ }$ μm and $h = 15.0{ }$ μm ($\overline{a} = 1.75$ and $Ca_{s} = 6.0 \times 10^{ - 4} )$. The capsule ruptures and releases its inner content.

At some conditions, the stress / deformation is too high and the capsule bursts, releasing its inner content. Figure 6 presents snapshots of the flow of a capsule from System #1, $D = 174.6{ }$ μm and $h = 15.0$ μm ($\overline{a} = 1.75$ and $Ca_{s} = 6.0 \times 10^{ - 4} )$, which is one of the systems that releases its inner phase as it passes through the capillary throat. The stress acting on the capsule shell is maximum near the tip and backend of the capsule when it is close to the minimum diameter place^37^. If the shell cannot sustain the imposed stress, it ruptures and the inner phase flows out of the capsule. In the case shown in Fig. 6, the inner content is released from the tip of the capsule, as it is clear in frame $104.290{\text{ s}}.$ In the last frame shown, e.g. $104.430{\text{ s}}$, the polymeric shell (almost fully emptied) and the dyed inner phase can be clearly observed. All capsules from System #1 presented the same behavior: they burst as the flowed through the constriction.

**Figure 7:**
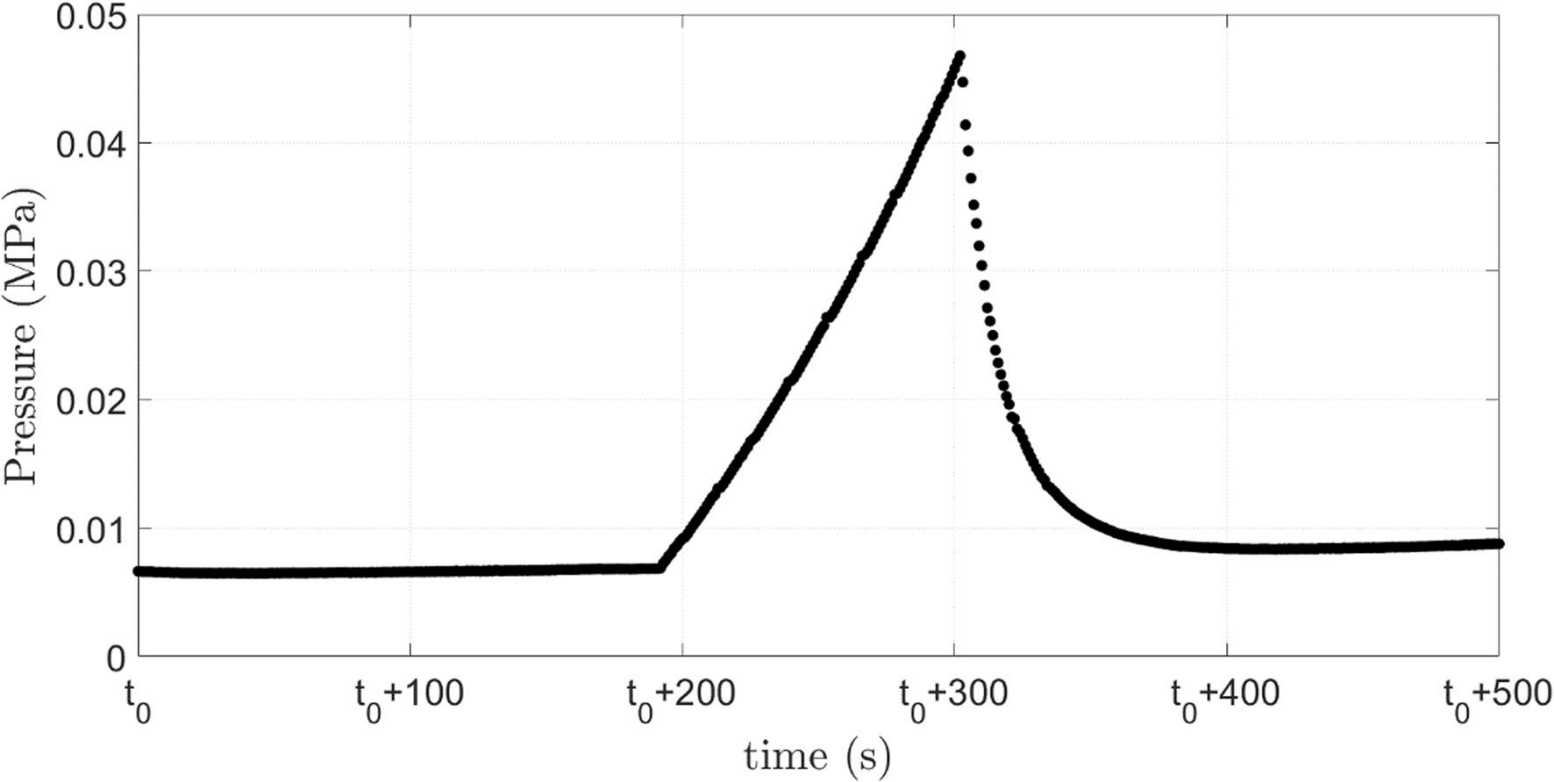
Evolution of the inlet pressure as the capsule flows through the constriction. System #1, $D = 174.6{ }$ μm and $h = 15{ }$ μm ($\overline{a} = 1.75$ and $Ca_{s} = 6.0 \times 10^{ - 4} )$.

Figure 7 presents the evolution of the inlet pressure for the flow shown in Fig. 6. The behavior is similar to the case at which the capsule did not rupture, presented in Fig. 2. The pressure rises as the capsule is deformed to flow through the capillary throat. Once the shell ruptures, flow resistance is decreased and the inlet pressure falls. It is important to note that the inlet pressure would be higher if the capsule shell have not ruptured.

**Figure 8:**
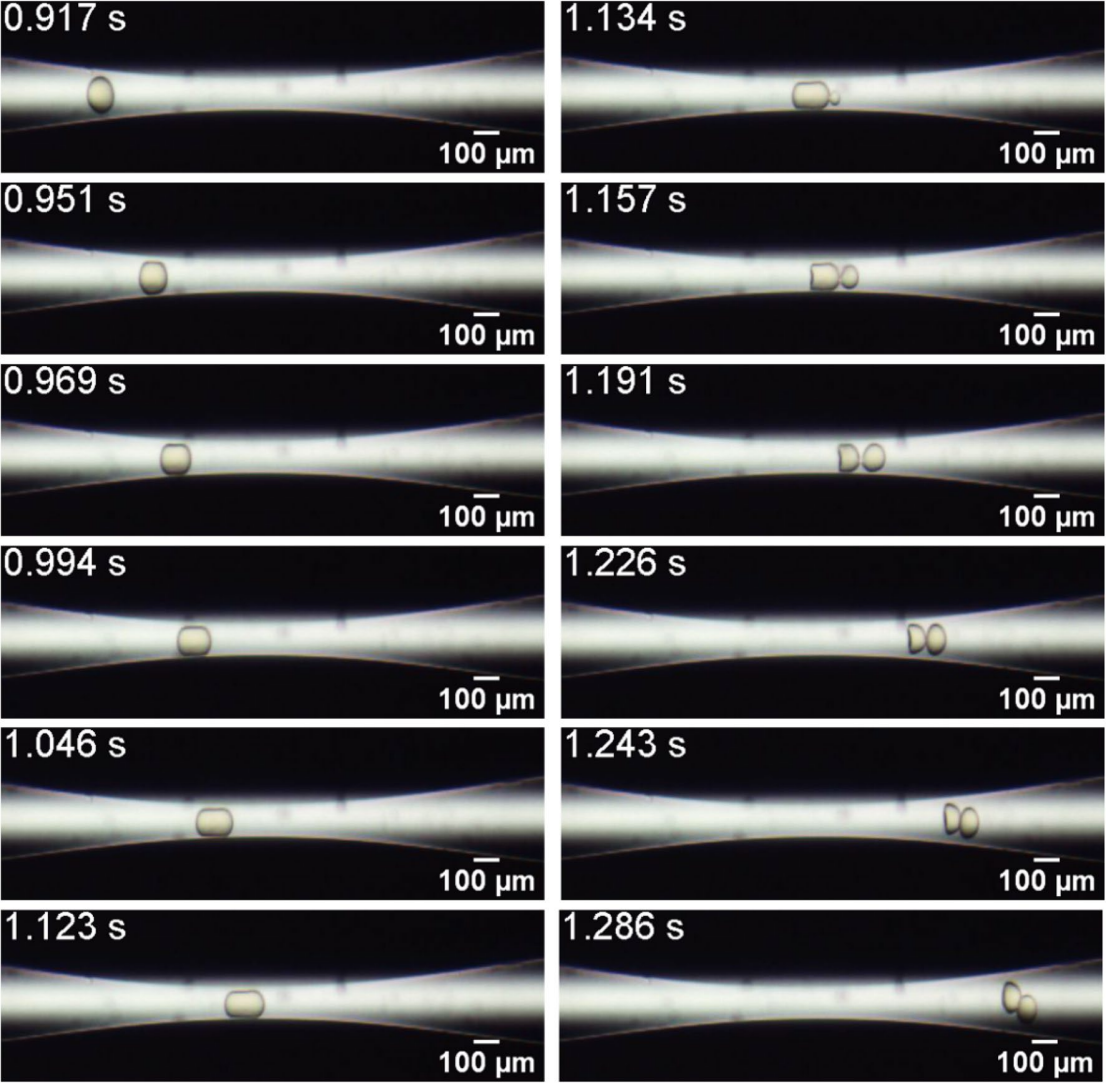
Evolution of the microcapsule position and configuration as it flows through the constriction. System #4, $D = 124.4{ }$ μm and $h = 5.6{ }$ μm ($\overline{a} = 1.75$ and $Ca_{s} = 6.0 \times 10^{ - 4} )$. The capsule ruptures and releases its inner content.

The capsule rupture and consequent inner phase release mechanism was similar for all the systems that were not strong enough to resist to the imposed stress as the capsule flows through the constriction. Figure 8 presents snapshots of the flow of a capsule from System #4, $D = 124.4{ }$ μm and $h = 5.6{ }$ μm ($\overline{a} = 1.24$ and $Ca_{s} = 1.6 \times 10^{ - 3} )$, which is another system that releases its inner phase from the tip of the capsule as it passes through the capillary throat.

Table 2 summarizes the averaged maximum extra pressure-drop and deformation along the flow direction associated with the flow of the capsule through the constriction and the state of the capsule after flowing through the constricted capillary for the 5 systems tested. As mentioned before, experiments were repeated for at least 5 capsules of each system. The flow behavior was the same for all capsules of a same system. Table 2 also presents the standard deviation of the maximum pressure for each system. For systems #4 and #5, the maximum extra pressure-drop was below the transducer resolution ($2.07{ } \times 10^{ - 3} {\text{ MPa}}$) and could not be measured.

**Table 2:** Maximum extra pressure-drop and state of the capsule after passing through the constriction for each system tested.

System ID	$D$ (μm)	$h$ (μm)	$Ca_{s}$	$\overline{a} = \frac{D}{{D_{c} }}$	$\Delta P$ (MPa)	$\varepsilon_{x} = \frac{{L_{x,max} }}{{L_{x,0} }}$	Ruptured
#1	174.6 ± 4.6	15.0 ± 2.1	$6 \times 10^{ - 4}$	1.75	42.7 ± 7.6 $\times 10^{ - 3}$	1.81	Yes
#2	143.8 ± 4.2	6.3 ± 1.5	$1.4 \times 10^{ - 3}$	1.44	4.97 ± 1.1 $\times 10^{ - 3}$	1.60	Yes
#3	144.4 ± 6.7	15.2 ± 3.7	$5.9 \times 10^{ - 4}$	1.44	34.8 ± 3.9 $\times 10^{ - 3}$	1.71	No
#4	124.4 ± 1.7	5.6 ± 1.0	$1.6 \times 10^{ - 3}$	1.24	< $2.07 \times 10^{ - 3}$	1.36	Yes
#5	109.1 ± 2.9	5.3 ± 1.1	$1.7 \times 10^{ - 3}$	1.09	< $2.07 \times 10^{ - 3}$	1.47	No

The effect of the capsule diameter, represented by $\overline{a}$, for a fixed shell thickness can be analyzed by comparing the flow behavior of capsules from systems #1 and #3 ($h\sim 15{ }$ μm) at $Ca_{s} \approx 6 \times 10^{ - 4}$, and capsules from systems #2, #4 and #5 ($h\sim 5 - 6{ }$ μm) at $Ca_{s} \approx 1.5 \times 10^{ - 3} .$ As expected, the inlet pressure rises with capsule diameter, since larger capsules need to deform more to flow through the capillary throat. The increase in the deformation / stress with the capsule’s diameter was enough to cause shell rupture.

Thicker shells lead to lower surface capillary number $Ca_{s}$, i.e. lower ratio between the viscous force, which drives deformation, and elastic force, which resists the deformation. Capsules that flow at lower surface capillary numbers are harder to deform and are also more resistant to rupture. This is clear by comparing the flow behavior of capsules from systems #2 and #3, which have the same diameter (*D* ~ 144 μm, $\overline{a} = 1.44$) and different shell thickness. Capsules from System #3, which have thicker shells and consequently lower surface capillary number, led to higher maximum inlet pressure and were able to flow through the constriction without releasing their inner phase.

**Figure 9:**
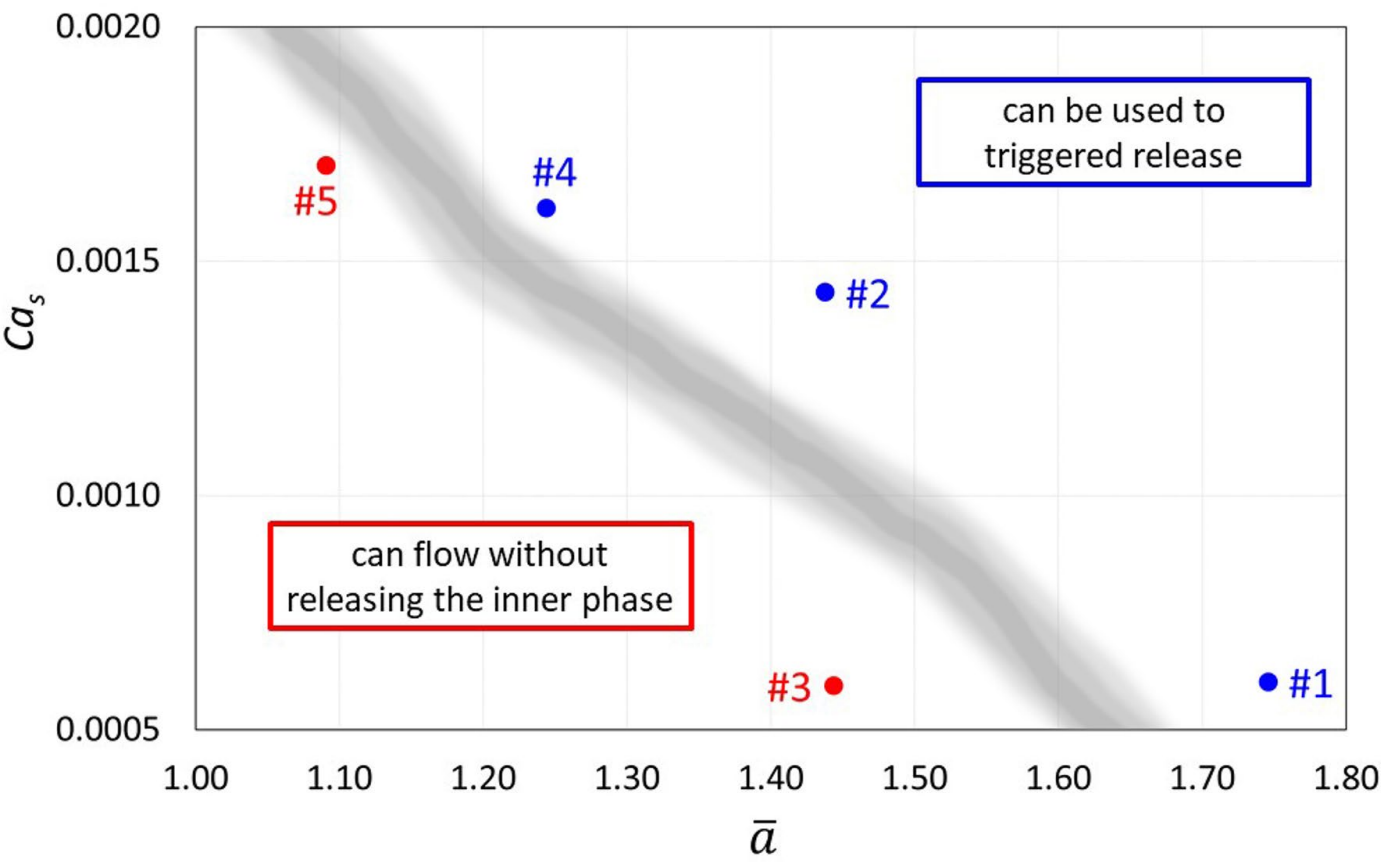
Map of state of capsule after flowing through the constriction as a function of dimensionless capsule diameter $\overline{a}$ and surface capillary number $Ca_{s}$: (red circle) reversible deformation and (blue circle) rupture.

Reversible deformation was only observed in microcapsules from systems #3 and #5, despite their differences in size and shell thickness. For a fixed capillary geometry and Reynolds number, the state of the capsule after flowing through the constriction, i.e. shell rupture or reversible deformation, is a function of the dimensionless capsule diameter $\overline{a}$ and surface capillary number $Ca_{s} .$ Figure 9 shows a map of the five systems tested in the parameter space $\overline{a} {\ } vs.\ Ca_{s}$. The capsules in the right-top portion of the plot, i.e. high capsule to throat diameter ratio and surface capillary number, rupture as they flow through the constriction. At a fixed capsule diameter, there is a critical surface capillary number above which the capsule bursts. This critical surface capillary number value decreases with capsule diameter. If the application requires capsules that release their content triggered by external stress caused by flow through constricted channels, the dimensionless parameters of the flow should be above the transition zone of Fig. 9; if the capsule is designed to change the flow mobility in porous media or require to flow through constricted passages without releasing their inner phase content, the flow dimensionless parameter should be in the region below the transition zone. Experiments with different constriction geometries, flow rate and shell material may enable the development of a general phase diagram-like plot that could be used to design capsules accordingly to their applications.

## Conclusions

The deformation dynamics of soft microcapsules as they flow through a constricted capillary strongly depends on the capillary geometry, capsule dimensions and properties and flow rate. If the stress imposed by the flow is higher than what the capsule shell can sustain, it will rupture and release its internal content. At a fixed capillary geometry and flow rate, the dynamics of the flow is a function of the ratio between the capsule diameter and capillary throat diameter, which defines the degree of deformation needed to flow the capsule through a constriction, and the surface capillary number, which represents the ratio of the viscous forces that deform the capsule and the elastic force that resists deformation.

Simultaneous flow visualization and pressure difference measurements enabled the detailed analysis of the flow and the effect of surface capillary number and dimensionless capsule diameter. The results included pressure difference, capsule speed and deformation data that can be used in the validation of different numerical models of the problem.

The results show that for the conditions explored, at each level of imposed deformation, that can be characterized by the capsule to constriction diameter ratio, there is a surface capillary number above which the membrane ruptures. The results should be used in the design of capsules for specific applications, i.e. if the release of their inner content should occur or not during the flow through constricted channels.

## Materials and methods

### Microcapsules production

Microcapsules were formed from O/W/O double emulsion templates, which were produced by microfluidics^38^. The three phases of the double emulsion template were: a refined commercial sunflower oil (Liza, Cargill Agricola S.A., Brazil) labeled with an orange food-grade dye as inner phase ($\mu_{i} = 55.3\,\, {\text{mPa}}\,{\text{s}}$); a mixture of 0.5 wt.% low-acyl gellan gum Kelcogel CG-LA (CP Kelco Brasil S/A, Brazil) and 2 wt.% polyoxyethylene sorbitan monolaurate, Tween 20 (Sigma-Aldrich, USA), in ultrapure water with resistivity 18.2 MΩ/cm (Direct-Q3 UV System, Millipore Co., USA) as middle phase; and a sunflower oil dispersion containing 1 wt.% calcium acetate (Sigma-Aldrich, USA) and 5 wt.% polyglycerol-polyricinoleate commercially named Grinstead PGPR super (Danisco Brasil, Brazil) as continuous phase.

Most biodegradable polymers are biocompatible and thus very attractive as shell materials, especially when the application involves interaction with living organisms and natural degradation of microcapsules. Gellan gum is a biocompatible polymer that produces stronger and less permeable gels than the largely used alginate-based ones. It is a linear, anionic, and high molecular weight exopolysaccharide secreted by the bacterium Sphingomonas elodea with some valuable characteristics such as malleability and mucoadhesive ability^39^. The gelation process of gellan starts with the formation of double helices generated from an initial disordered coil (chain ordering) followed by the connection of the double helices enabled by the presence of cations. The structures formed are thermo^40^ and pH-responsive^41^.

**Figure 10:**
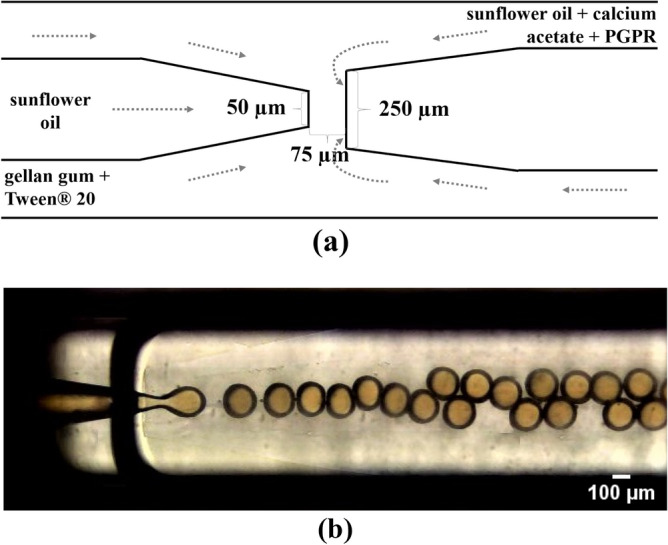
Three-dimensional coaxial microfluidic device used to produce gellan gum microcapsules. (**a**) Sketch of the microcapillary geometry for generating O/W/O templates and (**b**) Image of double emulsion production.

To produce monodisperse microcapsules, a three-dimensional coaxial microfluidic device (as sketched in Fig. 10) was attached to a glass slide^42^. It was made of two cylindrical glass-capillaries (World Precision Instruments Inc., USA), with inner and outer diameters of 0.58 mm and 1 mm nested into a square capillary with inner dimension of 1.05 mm (Atlantic International Technology Inc., USA). The tips of the cylindrical glass-capillaries were sanded to the final inner diameters, 50 μm for the injection capillary (left capillary in Fig. 10) and 250 μm for the collection capillary (right capillary in Fig. 10), after being tapered to an inner diameter of approximately 20 μm with a micropipette puller (model P-1000, Sutter Instrument Co., USA). The distance between them was fixed at 75 μm. The collection capillary was treated with a commercial rain repellent Glass Shield (Inove Pack do Brasil, Brazil) for 60 min to render a hydrophobic surface, while the injection capillary was treated with a polyelectrolytes solution composed of 1 wt.% poly(acrylamide-co-diallydimethyl-ammonium chloride) (Sigma-Aldrich, USA) and 2 mol/L NaCl for 60 min to render a hydrophilic surface. Stainless steel dispensing needles of inner and outer diameters 0.66 mm and 0.91 mm (model 304, McMaster-Carr, USA) were fixed at the junctions between capillaries or at their ends to enable fluids injection.

The inner phase was pumped through the injection capillary, while the middle and continuous phases were pumped in opposite directions through the interstices between the cylindrical and square capillaries. Then, all fluids were forced through the exit orifice of the collection tube (see Fig. 10). All flow rates were controlled by syringe-pumps (model Pump 11, Harvard Apparatus, USA). The O/W/O template formation was monitored using an inverted microscope (model DMi8, Leica Microsystem, Germany) equipped with a high-speed camera (model Fastcam SA-3, Photron, USA).

The microcapsules were collected in a glass vial with a small volume of hexane. Immediately after, acetate buffer (0.074 mol/L, pH 4.5) was added to the vial, the hexane excess containing the oil from the continuous phase was removed and the residual hexane was evaporated at room temperature for 24 h to ensure an oil-free capsular dispersion. The flow rates of the three phases were varied in order to produce microcapsules with different outer diameter $D$ and shell thickness $h$^37^. Microscope images of the microcapsules were captured with the inverted microscope model DMi8 (Leica Microsystems, Germany) and processed using the software Leica Application Suite X (Leica Microsystems, Germany) to determine the outer diameter $D$ and the shell thickness $h$ of the microcapsules. The shear modulus of the shell material was determined using a cantilevered-capillary force apparatus^43^; the measure value was $G = 10\,\,{\text{ kPa}}$.

### Flow through constriction

A constricted glass capillary with a diameter of D_0_ = 300 μm and constriction diameter equal to $D_{c} = 100$ μm, made by Hilgenberg (Germany), was used in the experiments. The constriction geometry was smooth, with a constriction length of $L_{c} = 4$ mm. It was treated with a polyelectrolytes solution composed of 1 wt% poly(acrylamide-codiallydimethyl-ammonium chloride) (Sigma-Aldrich, USA) and 2 mol/L NaCl to render a hydrophilic surface and then it was fixed on a microscope slide with Epoxy resin (Devcon Corp.,USA). All microcapsules have a diameter smaller than the glass capillary (300 μm) but larger than its constriction (100 μm).

**Figure 11:**
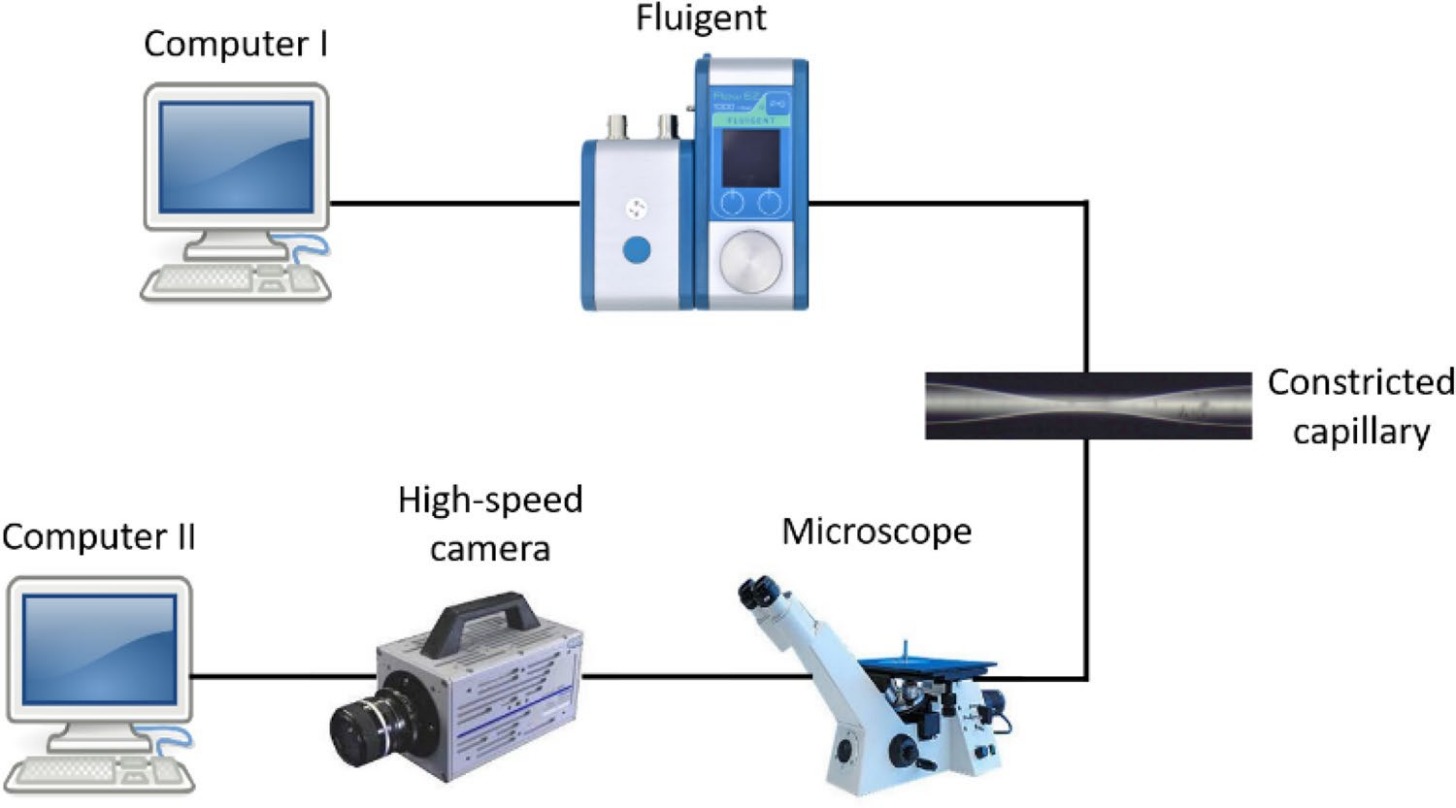
Scheme of the experimental set-up for flowing microcapsules through constricted capillaries.

A Fluigent system was used to feed the suspension through the capillary at constant flow rates and measure the inlet pressure response. It was composed of a stand-alone pressure pump (LineUp Flow EZ series—0 to 200 kPa) combined with a flow rate control unit (Flow Unit S), a Link module (LineUp) that connects the chain to a PC for software control and a 15 mL Falcon with a P-CAP air-tight connector where the injection fluid was kept. The Fluigent system mounted as described enables the control of the flow rate as well as of the dispense volume once the applied pressure automatically adjusts in the background to maintain the flow rate. The setup enables running experiments at fixed flow rates and measuring the injection pressure. An inverted microscope (model DMi8, Leica Microsystem, Germany) equipped with a high-speed camera (model Fastcam SA-3, Photron, USA) and a second computer were used to monitor and record real time images of microcapsules flow. Figure 11 shows a schematic representation of the experimental set-up.

The microcapsules were suspended in a saturated sucrose solution to increase the carrier fluid viscosity, which was fixed at $\mu_{o} = 11.5\,\, {\text{mPa}}\,{\text{s}},$ enabling pressure-drop measurements. Results were all obtained at a flow rate of $q = 0.2\,\,{\text{ ml}}/{\text{h}}$, which corresponded to an average velocity in the straight portion of the capillary of $\overline{V} = 4q/\pi D_{0}^{4} \approx 800$ μm/s.

### Image processing

All images extracted from the videos were processed with a MATLAB code based on frame difference to detect the moving capsules. The images were converted into grayscale and, after denoising, into binary images. Then, morphological operations were performed and small unwanted objects were removed from the background.

The length scale was determined from the known constriction diameter. The position of the tip of the microcapsules in the flow direction ($x$) and their length in the flow direction $\left( {L_{x} } \right)$ were measured in each frame. The velocity of the capsules was calculated from the distance covered over a set of frames and its corresponding time.

## Supplementary Information

Supplementary Video 1.

Supplementary Video 2.

## References

[1] Datta SS (2014). 25^th^ Anniversary Article: Double emulsion templated solid microcapsules: Mechanics and controlled release. Adv. Mater..

[2] Andrade B (2015). New frontiers for encapsulation in the chemical industry. ACS Appl. Mater. Interfaces.

[3] Amstad E (2017). Capsules: their past and opportunities for their future. ACS Macro Lett..

[4] Fang Z, Bhandari B (2010). Encapsulation of polyphenols: A review. Trends Food Sci. Tech..

[5] Burgain J, Gaiani C, Linder M, Scher J (2011). Encapsulation of probiotic living cells: From laboratory scale to industrial applications. J. Food Eng..

[6] Bakry, A. M. *et al.* Microencapsulation of oils: a comprehensive review of benefits, techniques, and applications. *Compr. Rev. Food Sci. Food Saf.* **15**(1), 143–182 (2015). 10.1111/1541-4337.12179 33371581

[7] Pena B, Panisello C, Areste G, Garcia-Valls R, Gumi T (2012). Preparation and characterization of polysulfone microcapsules for perfume release. Chem. Eng. J..

[8] Martins IM, Barreiro MF, Coelho M, Rodrigues AE (2014). Microencapsulation of essential oils with biodegradable polymeric carriers for cosmetic applications. J. Chem. Eng. J..

[9] De Cock JL (2010). Polymeric multilayer capsules in drug delivery. Angew. Chem. Int. Ed..

[10] Huang L, Zhou J, Chen Y, Li W, Han X, Wang L (2020). Engineering microcapsules for simultaneous delivery of combinational therapeutics. Adv. Mater. Technol..

[11] Gun WJ, Routh AF (2013). Microcapsule flow behavior in porous media. Chem. Eng. Sci..

[12] Abbaspourrad A (2013). Microfluidic fabrication of stable gas-filled microcapsules for acoustic contrast enhancement. Langmuir.

[13] Ribeiro, R. C. 3-D visualization of oil displacement in porous media by the injection of a microcapsule suspension using confocal microscopy. *SPE ATCE*, SPE-204265-STU, (2020).

[14] Zhao Y (2011). Enhanced encapsulation of actives in self-sealing microcapsules by precipitation in capsule shells. Langmuir.

[15] Chen PW, Erb RM, Studart AR (2011). Designer polymer-based microcapsules made using microfluidics. Langmuir.

[16] Dubey R, Shami T, Rao K (2009). Microencapsulation technology and applications. Def. Sci. J..

[17] Windbergs M, Zhao Y, Heyman J, Weitz DA (2013). Biodegradable core–shell carriers for simultaneous encapsulation of synergistic actives. J. Am. Chem. Soc..

[18] Sun BJ, Shum HC, Holtze C, Weitz DA (2010). Microfluidic melt emulsification for encapsulation and release of actives. ACS Appl. Mater. Interfaces.

[19] Abbaspourrad A, Datta SS, Weitz DA (2013). Controlling release from pH-responsive microcapsules. Langmuir.

[20] Zhang W (2017). Osmotic pressure triggered rapid release of encapsulated enzymes with enhanced activity. Adv. Funct. Mater..

[21] Zhang W (2019). Controllable fabrication of inhomogeneous microcapsules for triggered release by osmotic pressure. Small.

[22] Abbaspourrad A, Carroll NJ, Kim SH, Weitz DA (2013). Polymer microcapsules with programmable active release. J. Am. Chem. Soc..

[23] Kim SH, Kim JW, Cho JC, Weitz DA (2011). Double-emulsion drops with ultra-thin shells for capsule templates. Lab Chip.

[24] Freund JB (2014). Numerical simulation of flowing blood cells. Annu. Rev. Fluid Mech..

[25] Mao X, Huang T (2012). Exploiting mechanical biomarkers in microfluidics. Lab Chip.

[26] Suresh S (2007). Biomechanics and biophysics of cancer cells. Acta Biomater..

[27] Hou HW (2009). Deformability study of breast cancer cells using microfluidics. Biomed. Microdevices.

[28] Barthès-Biesel D (2016). Motion and deformation of elastic capsules and vesicles in flow. Annu. Rev. Fluid Mech..

[29] Leyrat-Maurin A, Barthes-Biesel D (1994). Motion of a seformable capsule through a hyperbolic constriction. J. Fluid Mech..

[30] Rorai C, Touchard A, Zhu L, Brandt L (2015). Motion of an elastic capsule in a constricted microchannel. Eur. Phys. J. E.

[31] Dimitrakopoulos P, Kuriakose S (2015). Determining a membrane's shear modulus, independent of its area-dilatation modulus, via capsule flow in a converging micro-capillary. Soft Matter.

[32] Risso F, Colle-Paillot F, Zagzoule M (2006). Experimental investigation of a bioartificial capsule flowing in a narrow tube. J. Fluid Mech..

[33] Lefebvre Y, Leclerc E, Barthès-Biesel D, Walter J, Edwards-Levy F (2008). Flow of artificial microcapsules in microfluidic channels: A method for determining the elastic -properties of the membrane. Phys. Fluids.

[34] Leclerc E, Kinoshita H, Fujji T, Barthès-Biesel D (2012). Transient flow of microcapsules through convergent-divergent microchannels. Microfluid. Nanofluid..

[35] Leong FY, Li Q, Lim CT, Chiam K (2011). H Modeling cell entry into a micro-channel. Biomec. Model. Mechanobiol..

[36] do Nascimento, D. F *et al*. Flow of tunable elastic microcapsules through constrictions. *Sci. Rep*. **7**, 11898 (2017). 10.1038/s41598-017-11950-2 PMC5605504 28928386

[37] Roca JF, Menezes IF, Carvalho MS (2021). Mobility reduction in the flow of an elastic microcapsule through a constricted channel. Ind. Eng. Chem. Res..

[38] Michelon M, Leopercio BC, Carvalho MS (2020). Microfluidic production of aqueous suspensions of gellan-based microcapsules containing hydrophobic compounds. Chem. Eng. Sci..

[39] Zia K (2018). Recent trends on gellan gum blends with natural and synthetic polymers: A review. Int. J. Bio. Macromol..

[40] Graham S, Marina PF, Blencowe A (2019). Thermoresponsive polysaccharides and their thermoreversible physical hydrogel networks. Carbohy. Polym..

[41] controlled release of metformin HCl (2014). Carbohy. Polym..

[42] Utada AS (2005). Monodisperse double emulsions generated from a microcapillary device. Science.

[43] Huang, Y. H., Salmon, F., Kamble, A., Xu, A. X., Michelon, M., Leopercio, B. C., Carvalho, M. S. & Frostad, J. M. Methods and models for the mechanical characterization of edible microcapsules. Submitted to *Food Hydrocol..* (2021).

